# Carbamylation and glycation compete for collagen molecular aging *in vivo*

**DOI:** 10.1038/s41598-019-54817-4

**Published:** 2019-12-04

**Authors:** Camille Nicolas, Stéphane Jaisson, Laëtitia Gorisse, Frédéric J. Tessier, Céline Niquet-Léridon, Philippe Jacolot, Christine Pietrement, Philippe Gillery

**Affiliations:** 1 University of Reims Champagne-Ardenne, Laboratory of Biochemistry and Molecular Biology, CNRS/URCA UMR N° 7369 MEDyC, Reims, France; 2 University Hospital of Reims, Department of Pediatrics (Nephrology unit), Reims, France; 3 University Hospital of Reims, Laboratory of Pediatric Biology and Research, Reims, France; 4 University of Lille, CHU Lille, Inserm U995 - LIRIC - Lille Inflammation Research International Center, Lille, France; 5 Institut Polytechnique UniLaSalle, “Transformations & Agro-ressources” Unit, Beauvais, France

**Keywords:** Chemical modification, Proteins

## Abstract

Tissue aging is a complex phenomenon involving molecular aging of matrix proteins, which mainly results from their progressive alteration by nonenzymatic post-translational modifications (NEPTMs) such as glycation and carbamylation. These two reactions, which correspond to the binding of reactive metabolites (*i.e*. reducing sugars and urea-derived cyanate, respectively) on amino groups of proteins, occur during aging and are amplified in various chronic diseases such as diabetes mellitus or chronic renal disease (CKD). Since these reactions target the same functional groups, they can reciprocally compete for protein modification. Determining which NEPTM is predominant in tissues is necessary to better understand their role in the development of long-term complications of chronic diseases. For that purpose, two different murine models were used for reproducing such a competitive context: a CKD-diabetic mice model and a cyanate-consuming mice model. The competition has been evaluated by quantifying glycation and carbamylation products by LC-MS/MS in skin and aorta total extracts as well as in skin type I collagen. The results showed that the simultaneous enhancement of glycation and carbamylation reactions resulted in a decrease of the formation of glycation products (especially Amadori products) whereas the concentrations of homocitrulline, a carbamylation product, remained similar. These results, which have been obtained in both tissues and in purified skin type I collagen, suggest that carbamylation takes precedence over glycation for the modification of tissue proteins, but only in pathological conditions favouring these two NEPTMs. While glycation has been considered for a long time the predominant NEPTM of matrix proteins, carbamylation seems to also play an important role in tissue aging. The existence of competition between these NEPTMs must be taken into account to better understand the consequences of molecular aging of matrix proteins in tissue aging.

## Introduction

During their lifespan in the organism, proteins are exposed to various metabolic stresses that progressively alter their structural and functional properties. This continuous process, which corresponds to a molecular aging, is mainly due to various nonenzymatic posttranslational modifications (NEPTMs) which usually consist of the spontaneous reactions of metabolites with functional groups of proteins^1^. Among these modifications, glycation is one of the most studied and is characterized by the binding of sugars (or their by-products) to amino groups of proteins, followed by complex molecular rearrangements resulting in the formation of advanced glycation end products (AGEs), such as N^ε^-carboxymethyllysine (CML). Another reaction of growing interest is carbamylation, which results from the nonenzymatic reaction of cyanate (which mainly derives from urea dissociation) to protein amino groups. The most characteristic carbamylation-derived product (CDP) is homocitrulline (HCit), which is formed when cyanate reacts with ε-amino groups of lysine residues^2^.

These reactions occur during aging and are amplified upon several pathological contexts (*e.g*. diabetes mellitus or chronic kidney disease (CKD)) due to increased formation of reactive metabolites. Data from the literature have already reported that glycated and carbamylated proteins accumulate in tissues which are mainly constituted of long half-lived matrix proteins such as collagens and elastin^3–6^. The accumulation of modified matrix proteins may lead to a progressive impairment of tissue mechanical properties. For example, cross-links generated by collagen glycation are responsible for alterations of skin elastic properties^7^ and for an increase of tendon stiffness^8^, whereas carbamylated collagen fails to correctly associate into fibers^9^. In addition, modified matrix proteins are able to dysregulate various cell functions^9–11^.

However, protein molecular aging is a very complex phenomenon: NEPTMs cannot be considered separately since they simultaneously occur on all types of proteins in a non-regulated manner. Moreover, even though the theoretical number of available residues susceptible to modifications by NEPTMs is high, only a small proportion of them, sharing common characteristics (*e.g*. pKa, steric hindrance) are preferentially targeted, suggesting that different NEPTMs may compete for protein modification. We have previously shown that carbamylation and glycation compete for the modification of blood proteins, like hemoglobin or serum proteins, and that carbamylation takes precedence over glycation^12^.

Determining which reaction is predominant in tissues is of particular interest to better understand the role of these processes in the development of long-term complications of chronic diseases and to determine which reaction should be first targeted in potential therapeutic strategies. To address this question, the present study has investigated the competition between these two reactions in tissues by quantifying glycation and carbamylation products in skin and aorta extracts using murine models of diabetes and/or CKD.

## Methods

### Murine models

Nine-week-old diabetic *db/db* [C57Bl/KsJ-*db/db*] mice and the corresponding controls (*db/ + *) were obtained from Janvier (Saint Berthevin, France). Animals were fed *ad libitum* and housed in plastic cages with sawdust-covered flooring, in a room with constant ambient temperature and a 12-hour light-dark cycle. All animal procedures were conducted in accordance with French government policies (Services Vétérinaires de la Santé et de la Production Animale, Ministère de l’Agriculture), and this study was approved by the institutional animal care committee (“Comité d’éthique en expérimentation animale de Reims Champagne Ardenne”, registration 56).

**Figure 1:**
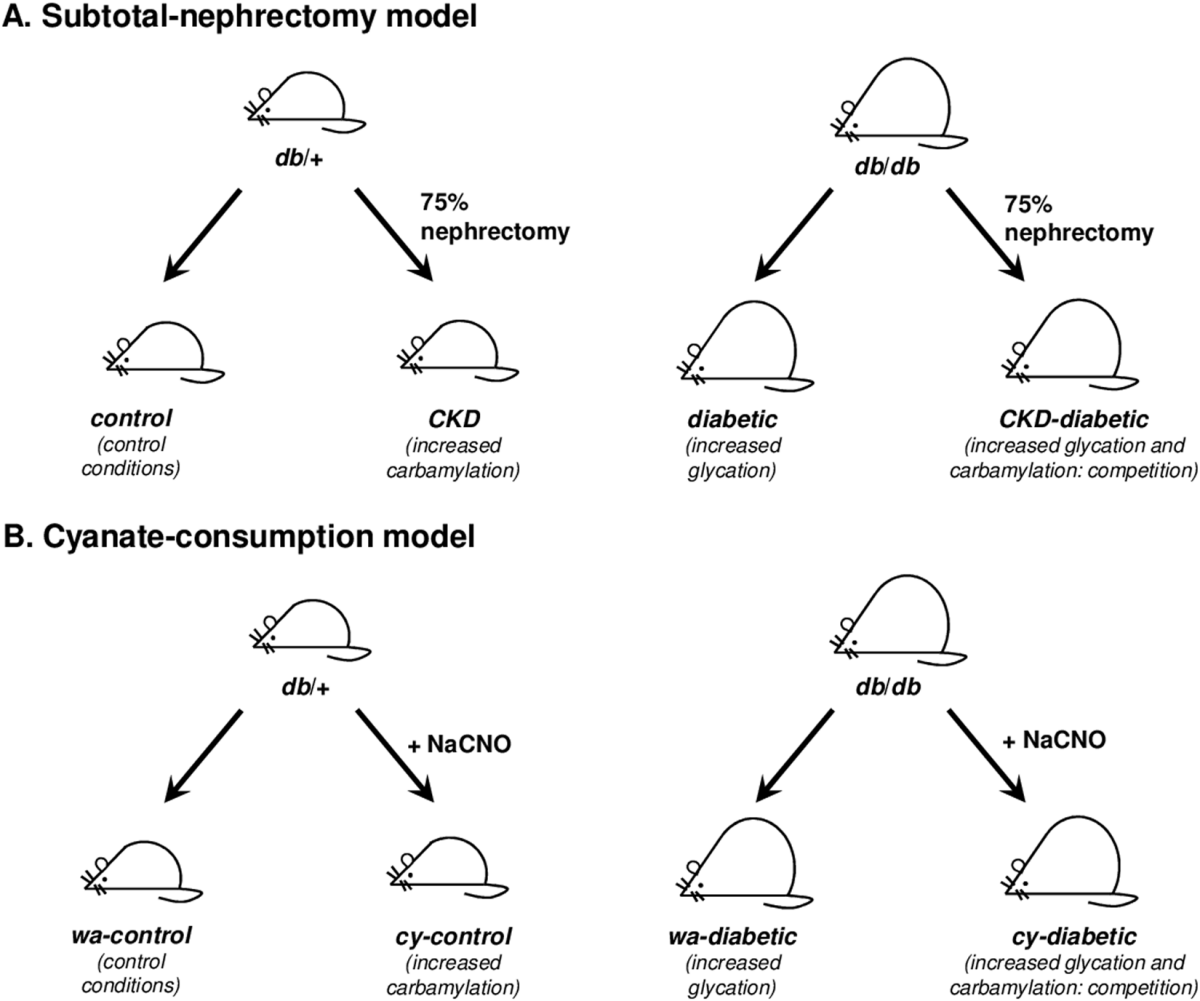
Schematic representation of the different mice models used for evaluating the competition between glycation and carbamylation *in vivo*. *Subtotal nephrectomy model* (**A**): *db/db* and *db/ + *mice were subjected to a two-step subtotal-nephrectomy procedure used to induce CKD: half of the left kidney was removed by resection of the poles, and bleeding was controlled by electrocoagulation, and one week later, the right kidney was removed, leading to a 75% reduction of total renal mass and a chronic hyperuremia. Control animals underwent sham operations. n = 6 for each group. Abreviations: CKD: chronic kidney disease. *Cyanate-consumption model* (**B**): *db/db* and *db/ + *mice received regular drinking water or drinking water containing 1 mM sodium cyanate ( + NaCNO). n = 6 for each group. Abreviations: wa: regular water, cy: water containing sodium cyanate.

Competition between glycation and carbamylation was evaluated using different combinations of murine models. Diabetic (*db/db*) mice were used to reproduce hyperglycation conditions. Subtotal nephrectomy or addition of cyanate to drinking water were used to enhance carbamylation as seen in CKD. Each experiment was conducted using the following groups (Fig. 1):

- Control conditions: non diabetic *db*/ + mice without nephrectomy (control group, Fig. 1A) or fed with regular water (wa-control groups, Fig. 1B).
- Increased carbamylation: *db/ + *mice with subtotal nephrectomy (CKD group, Fig. 1A) or fed with cyanate-supplemented water (cy-control group, Fig. 1B).
- Increased glycation: *db/db* mice (diabetic (Fig. 1A) or wa-diabetic groups (Fig. 1B)).
- Increased glycation and carbamylation ( = competition): *db/db* mice with subtotal nephrectomy (CKD-diabetic group, Fig. 1A) or fed with cyanate-supplemented water (cy-diabetic group, Fig. 1B).

*Subtotal-nephrectomy model: db/db* and *db/ + *mice (n = 6 per group) were randomly assigned to CKD groups (CKD-diabetic group, CKD group) or control groups (diabetic group, control group). Surgery was performed under isoflurane anaesthesia. A two-step subtotal-nephrectomy procedure was used to induce CKD: (i) half of the left kidney pole was removed by incision, and bleeding was controlled by electrocoagulation, (ii) one week later, the right kidney was removed, leading to a 75% reduction of total renal mass and a chronic hyperuremia. Control animals underwent sham operations. Five weeks after surgery, mice were euthanized under xylazine (Rompun 2%; Bayer, Leverkusen, Germany) and ketamine (Clorketam 1000; Vetoquinol SA, Lirre, France) (60 and 120 μg/g of body weight, respectively) anaesthesia, and blood was collected in heparin-containing tubes by cardiac puncture. Skin, abdominal aorta and tail were collected. Hematocrit was immediately analyzed and plasma and tissues were stored at −80 °C until analysis.

*Cyanate-consumption model: db/db* and *db/ + *mice (n = 6 per group) were randomly assigned to four groups that received normal drinking water (wa-control group and wa-diabetic group) or drinking water containing 1 mM sodium cyanate (cy-control group and cy-diabetic group) with the renewal of sodium cyanate-containing water twice a week. After six weeks, mice were euthanized, then blood and tissues were collected as described above.

### Blood measurements

Before anaesthesia, capillary glycemia was measured from a droplet obtained after tail incision, using a blood glucose monitoring system (Accu-Check®, Roche Diagnostics, Meylan, France). Urea was measured in plasma using a Cobas® analyzer (Roche Diagnostics). Hematocrit was determined by centrifuging heparinized blood in microcapillary tubes.

### Preparation of tissue extracts

Skin and aorta samples (100 mg) were placed in Lysing Matrix D® microtubes (MP Biomedicals) containing 1 mL 0.5 M acetic acid. The homogenization was performed using the FastPrep-24® System (MP Biomedicals) by applying 4 cycles of 40 s at a 6 m/s speed. After homogenization, 50 µg pepsin were added to the samples before an incubation for 24 h at 37 °C. After centrifugation (5 min, 24,000 g), the supernatant was stored at −80 °C until analysis.

### Extraction of type I collagen

Type I collagen was extracted from skin as previously described^5^. Briefly, skin samples were homogenized using FastPrep-24 system in 0.5 M acetic acid containing 1‰ (w/w) pepsin and incubated in this solution for 24 h at 4 °C, before precipitation with 0.7 M NaCl. Precipitates were solubilized with 18 mM acetic acid and dialyzed against distilled water for 3 days at 4 °C. Samples were then freeze-dried and stored at −80 °C until analysis.

### Quantification of carbamylation and glycation products

HCit being the most characteristic carbamylation-derived product, has been chosen for evaluating the extent of protein carbamylation^2^. As glycation reaction is a more complex process involving early and late steps, two different glycation products have been quantified in order to evaluate each phase of the process. Thus, furosine concentrations provided information about the formation of Amadori products (furosine is a by-product formed from fructose-lysine during acid hydrolysis) during the early steps of glycation, whereas CML provided information about the later steps (formation of AGEs) involving oxidative reactions^13^.

All samples (skin and aorta extracts, skin and tail type I collagen) were subjected to acid hydrolysis with 6 M hydrochloric acid for 18 h at 110 °C. Hydrolysates were evaporated to dryness twice under a nitrogen stream. Furosine, CML and HCit were then quantified by liquid chromatography coupled to tandem mass spectrometry (LC-MS/MS) as previously described^12^.

### Statistical analyses

Data were expressed as means ± standard deviations when described in the text and were represented as box plots in figures in order to better appreciate the dispersion of values. In box plots, the error bars represent minimum and maximum values, the horizontal lines indicate median values, and the extremities of the boxes indicate interquartile ranges. Statistical significances were evaluated using a two-tailed non-parametric Mann-Whitney’s U test. Differences were considered statistically significant for p-values < 0.05.

## Results

### Characterization of animal models

The different murine models used to study competition between glycation and carbamylation have been detailed in the Methods section and in Fig. 1. Body mass and different blood parameters were evaluated in order to validate the models before quantification of glycation and carbamylation products in skin and aorta (Table 1). In the subtotal nephrectomy model, CKD mice exhibited elevated urea concentrations in comparison with control group (19.5 ± 5.0 mmol/L *vs* 8.1 ± 1.4 mmol/L, p < 0.01) whereas hematocrit was significantly lower (37 ± 3% *vs* 44 ± 2%, p < 0.01), confirming the development of CKD. Diabetic and CKD-diabetic mice were overweighted in comparison with control ones (37.6 ± 6.0 g and 45.6 ± 3.2 g, respectively, *vs* 29.7 ± 2.0 g in control group, p < 0.05) and had increased glycemia (20.2 ± 4.3 mmol/L and 14.7 ± 10.9 mmol/L, respectively, *vs* 6.3 ± 0.8 mmol/L in control group, p < 0.01). The CKD-diabetic group had higher urea concentration when compared with the diabetic group (28.1 ± 7.9 mmol/L *vs* 9.3 ± 2.1 mmol/L, p < 0.01).

**Table 1:** Characteristics of the two mice models: CKD-diabetic mice and diabetic mice consuming cyanate.

	control	CKD	Diabetic	CKD-diabetic
Body mass (g)	29.7 ± 2.0	25.7 ± 2.5^**^	37.6 ± 6.0^*^	45.6 ± 3.2 ^ns^
Glucose (mmol/L)	6.3 ± 0.8	6.9 ± 1.5 ^ns^	20.2 ± 4.3^**^	14.7 ± 10.9 ^ns^
Hematocrit (%)	44 ± 2	37 ± 3^**^	43 ± 4 ^ns^	37 ± 5 ^ns^
Urea (mmol/L)	8.1 ± 1.4	19.5 ± 5.0^**^	9.3 ± 2.1 ^ns^	28.1 ± 7.9^‡‡^
	**wa-control**	**cy-control**	**wa-diabetic**	**cy-diabetic**
Body mass (g)	23.9 ± 1.4	28.4 ± 1.8 ^ns^	38.2 ± 3.1^##^	40.9 ± 4.6 ^ns^
Glucose (mmol/L)	6.4 ± 1.1	5.9 ± 1.2 ^ns^	18.5 ± 4.4^##^	22.2 ± 3.9 ^ns^
Hematocrit (%)	39 ± 4	42 ± 3 ^ns^	39 ± 2 ^ns^	42 ± 3 ^ns^
Urea (mmol/L)	8.1 ± 1.5	7.7 ± 1.6 ^ns^	13.7 ± 3.0^##^	10.6 ± 1.9^†^

control: *db/* + sham-operated mice; CKD: *db/* + mice with subtotal-nephrectomy; diabetic: *db/db* sham-operated mice; CKD-diabetic: *db/db* mice with subtotal-nephrectomy. CKD and diabetic groups were compared to control group (^*^p < 0.05, ^**^p < 0.01) and CKD-diabetic group was compared to diabetic group (^‡‡^*p* < 0.01), ^ns^:no significant difference.

wa-control: *db/* + mice fed with water; cy-control: *db/* + mice fed with water containing 1 mM sodium cyanate; wa-diabetic: *db/db* mice fed with water; cy-diabetic: *db/db* mice fed with water containing 1 mM sodium cyanate. cy-control and wa-diabetic groups were compared to control group (^##^*p* < 0.01) and cy-diabetic group was compared to wa-diabetic group (^†^*p* < 0.05), ^ns^:no significant difference.

In the cyanate-consuming model, wa- and cy-diabetic mice were overweighted, as expected, and no statistical differences in hematocrit values were observed between the different groups.

Diabetic mice drinking standard water (wa-diabetic) and diabetic mice drinking water supplemented with cyanate (cy-diabetic) had elevated blood glucose concentrations: 18.5 ± 4.4 mmol/L and 22.2 ± 3.9 mmol/L, respectively. Both concentrations were significantly (p < 0.01) increased in comparison with the control group (wa-control, 6.4 ± 1.1 mmol/L).

### Competition between glycation and carbamylation in skin and aorta extracts in the subtotal-nephrectomy model

**Figure 2:**
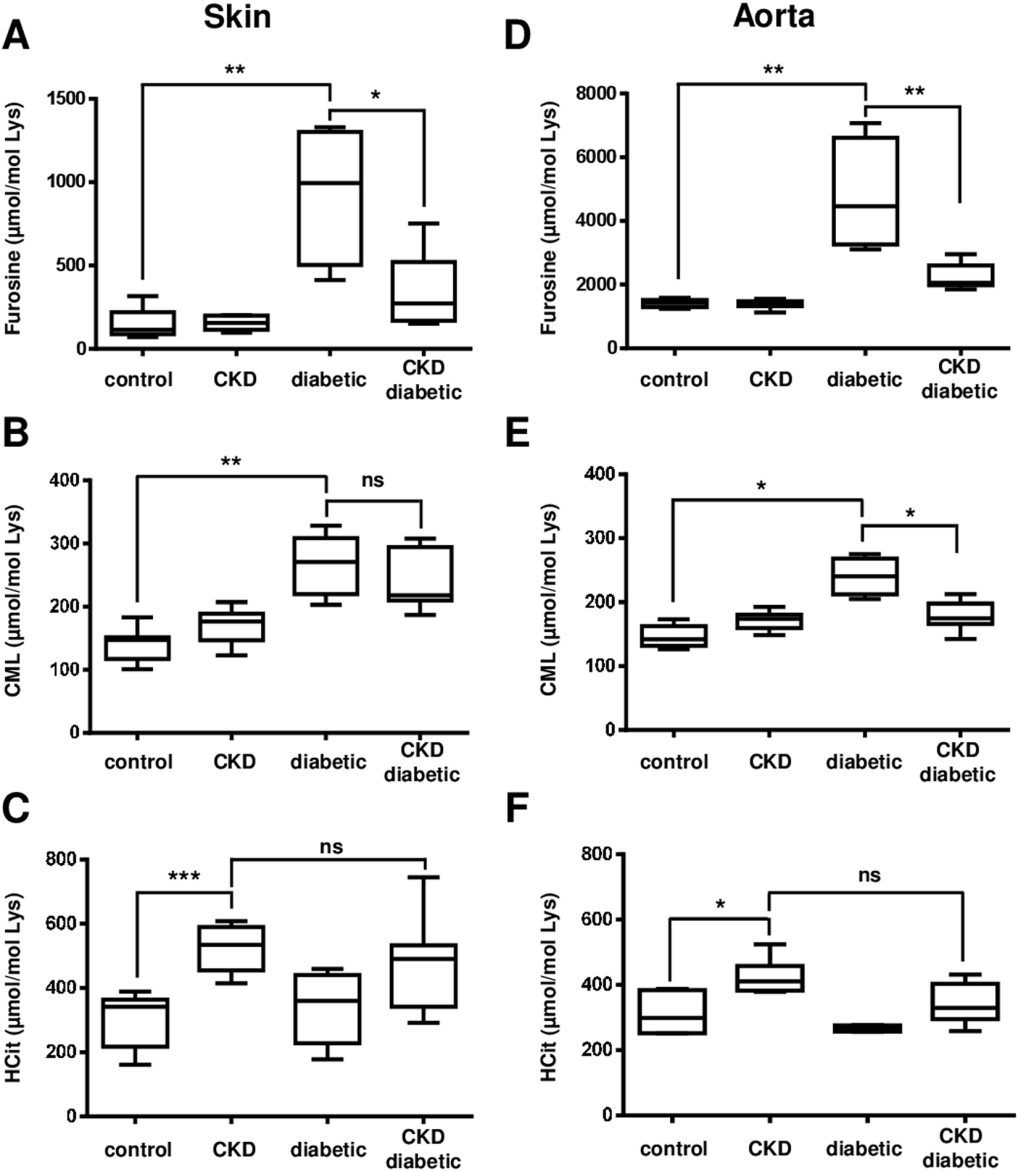
Competitive effects between glycation and carbamylation in skin and aorta in the subtotal-nephrectomy murine model. Nine week-old *db/ + *and *db/db* mice were subjected or not (control and diabetic groups) to a subtotal nephrectomy in order to induce CKD (CKD and CKD-diabetic groups). Five weeks after surgery, mice were euthanized and tissues were collected. Whole protein extracts were prepared from skin (**A–C**) and aorta (**D–F**) samples and submitted to acid hydrolysis for LC-MS/MS quantification of furosine (**A,D**), CML (**B,E**) and HCit (**C,F**). Results are presented as boxplots, in which the error bars represent minimum and maximum values, the horizontal lines indicate median values, and the extremities of the boxes indicate interquartile ranges. Data were compared using the Mann-Whitney’s U test: *p < 0.05, **p < 0.01, ***p < 0.001, ns: not significant.

*Carbamylation vs glycation:* in order to explore the potential competitive effect of carbamylation on glycation, two glycation products (*i.e*. furosine and CML) were quantified in each group and the interpretation of results focused on the differences of concentrations between diabetic and CKD-diabetic mice. In skin extracts, both markers were significantly (p < 0.01) increased in diabetic mice in comparison with control ones (Fig. 2A,B), whereas in CKD-diabetic mice, skin furosine concentrations were deeply reduced (−61%, p < 0.05) in comparison with diabetic mice (366 ± 217 µmol/mol Lys *vs* 933 ± 422 µmol/mol Lys, respectively). In contrast, no significant decrease of CML concentrations was observed.

In aorta extracts, the concentrations of the two glycation products were significantly (p < 0.01) more important in diabetic mice than in control ones, as observed in skin extracts (Fig. 2D,E). In CKD-diabetic mice, they were significantly lower than in diabetic mice (4778 ± 1774 µmol/mol Lys *vs* 2241 ± 410 µmol/mol Lys, p < 0.01 for furosine; 240 ± 29 µmol/mol Lys *vs* 179 ± 23 µmol/mol Lys, p < 0.05 for CML). These data suggest that carbamylation exerts a competitive effect on glycation in skin and aorta.

*Glycation vs carbamylation:* the competitive effect of glycation on carbamylation was evaluated by quantification of HCit in skin and aorta extracts and analysis of differences between CKD and CKD-diabetic groups. As expected, HCit content in skin and aorta extracts was significantly (p < 0.001) increased in CKD mice in comparison with control ones (Fig. 2C,F). The comparison of HCit concentrations between CKD and CKD-diabetic groups did not show any significant difference in skin as well in aorta, demonstrating that glycation does not interfere significantly with the carbamylation process.

### Competition between glycation and carbamylation in skin and aorta extracts in the cyanate-consumption model

To confirm results obtained in CKD-diabetic mice, similar experiments were performed using diabetic mice drinking water containing 1 mM cyanate. Although this model is less physiologically relevant than the subtotal nephrectomy model, its advantage is to reproduce the competitive conditions without side effects related to CKD, such as an increased oxidative stress which could participate in CML formation.

**Figure 3:**
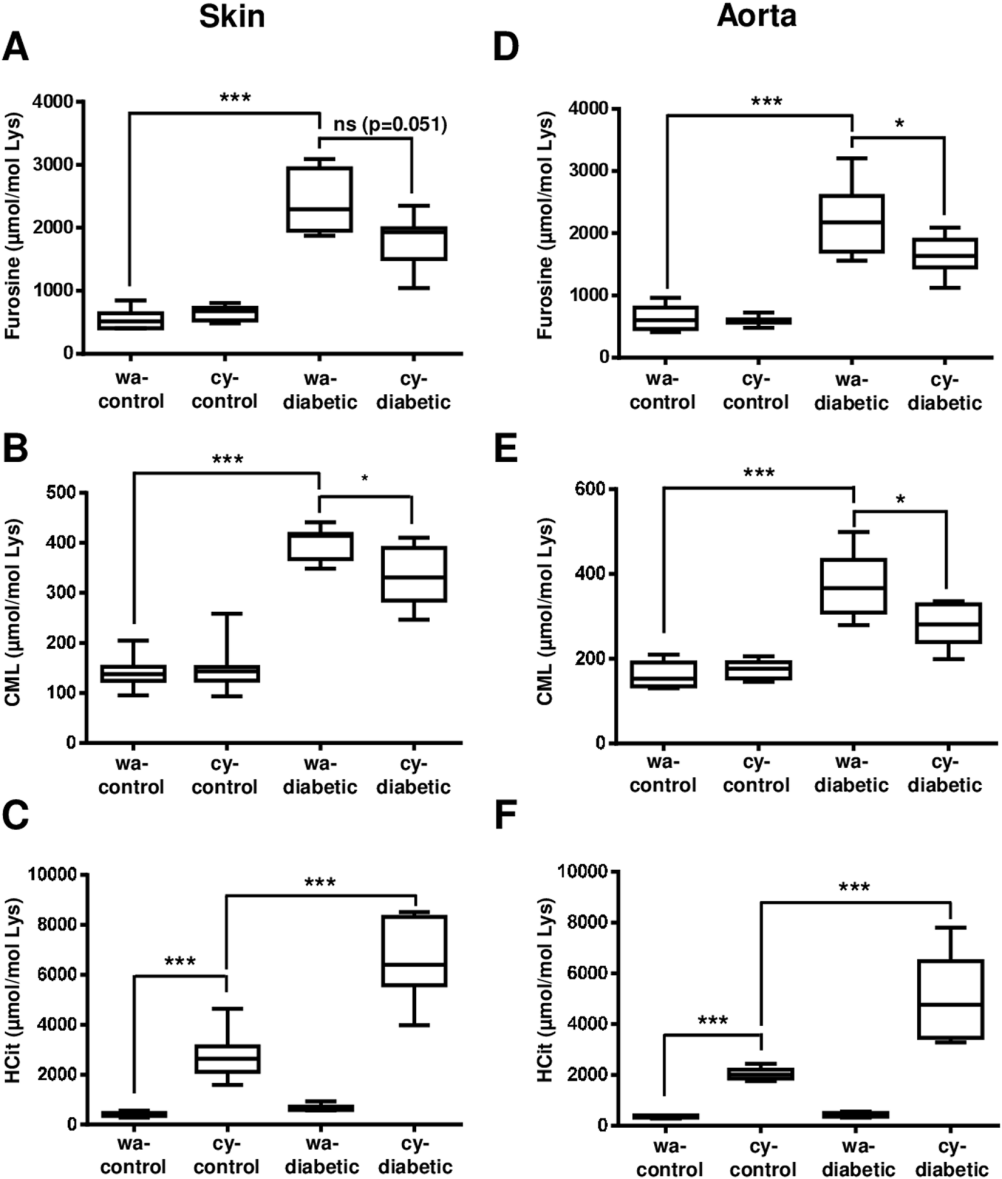
Competitive effects between glycation and carbamylation in skin and aorta in the cyanate-consuming murine model. Nine week-old *db/ + *and *db/db* mice received normal water (wa-control and wa-diabetic groups) or with water containing 1 mM sodium cyanate (cy-control and cy-diabetic groups). After six weeks of alimentation with sodium cyanate, mice were euthanized and tissues were collected. Whole protein extracts were prepared from skin (**A–C**) and aorta (**D–F**) samples and submitted to acid hydrolysis for LC-MS/MS quantification of furosine (**A,D**), CML (**B,E**) and HCit (**C,F**). Results are presented as boxplots, in which the error bars represent minimum and maximum values, the horizontal lines indicate median values, and the extremities of the boxes indicate interquartile ranges. Data were compared using the Mann-Whitney’s U test: *p < 0.05, ***p < 0.001, ns: not significant.

*Carbamylation vs glycation:* in skin extracts, furosine and CML concentrations were significantly (p < 0.001) increased in diabetic mice in comparison with control ones, as expected (Fig. 3). The addition of cyanate in drinking water of diabetic mice (cy-diabetic) decreased by 27% the furosine content (2461 ± 494 µmol/mol Lys for wa-diabetic mice vs 1785 ± 402 µmol/mol Lys for cy-diabetic mice), this decrease reaching almost significance (p = 0.051). A similar decrease was observed in CML concentrations (−16%, p < 0.05, 400.5 ± 32.1 µmol/mol Lys for wa-diabetic mice *vs* 336.3 ± 57.5 µmol/mol Lys for cy-diabetic mice) (Fig. 3A,B). The same pattern was observed in aorta extracts, the inhibition being about −26% (p < 0.05) for both markers (Fig. 3D,E).

*Glycation vs carbamylation:* like in the case of the subtotal nephrectomy model, no competition of glycation on carbamylation was observed (Fig. 3C,F). HCit concentrations were significantly (p < 0.001) increased in cy-control mice in comparison with wa-control mice in both tissues. The comparison between cy-diabetic and cy-control mice showed that HCit was not reduced but was even more important in the cy-diabetic conditions (2.4 fold increase in both skin and aorta, p < 0.01, Table 2).

**Table 2:** Reciprocal inhibitory effects between glycation and carbamylation reactions.

Subtotal-nephrectomy model	*carb. vs glyc.*		*glyc. vs carb.*
	Furosine	CML	HCit
Skin	−61%**	−8%^ns^	−14%^ns^
Aorta	−53%**	−26%*	−10%^ns^
Skin collagen	−44%*	+1%^ns^	−19%^ns^
**Cyanate-consuming model**	***carb. vs glyc***.		***glyc. vs carb***.
	**Furosine**	**CML**	**HCit**
Skin	**−27%**^**p=0.051**^	−16%*	+240%***
Aorta	−26%*	−26%*	+159%***
Skin collagen	−25%*	−2% ^ns^	+345%***

Competitive effect of carbamylation on glycation (*carb. vs glyc*.): mean concentrations of furosine and CML in CKD-diabetic or cy-diabetic groups were compared with those of diabetic or wa-diabetic groups, respectively. Competitive effect of glycation on carbamylation (*glyc. vs carb*.): mean concentrations of HCit concentrations in CKD groups or cy-control were compared with those of CKD-diabetic or cy-diabetic groups, respectively. Significant inhibitory effects were represented in bold font. Data were compared using the Mann-Whitney’s U test (*p < 0.05, **p < 0.01, ***p < 0.001, ns: not significant).

### Competition between glycation and carbamylation for type I collagen modifications

In order to determine the competition between glycation and carbamylation for the modifications of a typical matrix protein, type I collagen was extracted from skin samples in both models. It was not possible to extract collagen from aortas because of the limited sample size.

**Figure 4:**
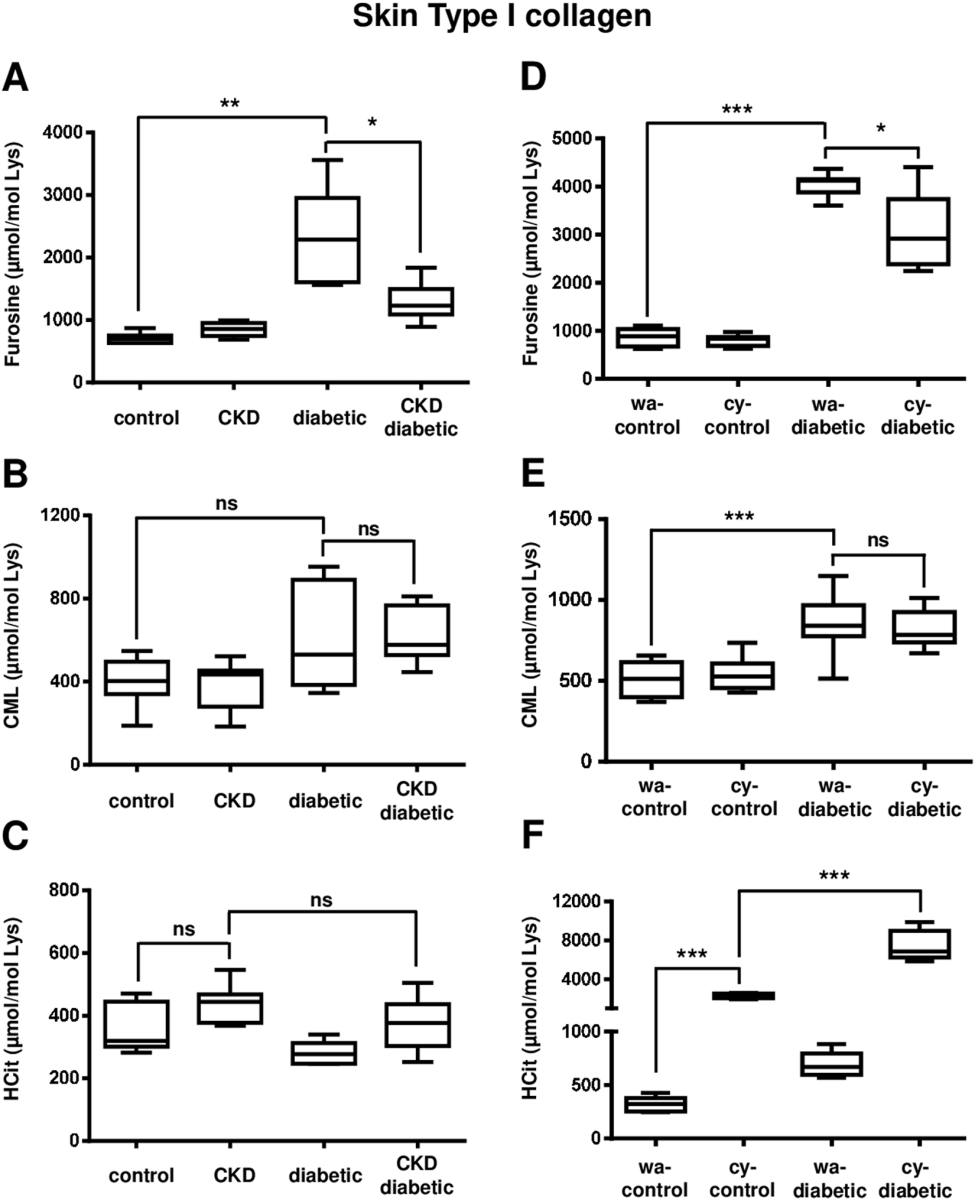
Competitive effects between glycation and carbamylation on modifications of skin type I collagen. Nine week-old *db/ + *and *db/db* mice were subjected or not (control and diabetic groups) to a subtotal nephrectomy in order to induce CKD (CKD and CKD-diabetic groups). Five weeks after surgery, mice were euthanized and tissues were collected (**A**–**C**). Nine week-old *db/ + *and *db/db* mice received normal water (wa-control and wa-diabetic groups) or with water containing 1 mM sodium cyanate (cy-control and cy-diabetic groups). After six weeks of alimentation with sodium cyanate, mice were euthanized and tissues were collected (**D**–**F**). Type I collagen was extracted from skin samples and submitted to acid hydrolysis for LC-MS/MS quantification of furosine (**A,D**), CML (**B,E**) and HCit (**C,F**). Results are presented as boxplots, in which the error bars represent minimum and maximum values, the horizontal lines indicate median values, and the extremities of the boxes indicate interquartile ranges. Data were compared using the Mann-Whitney’s U test: *p < 0.05, **p < 0.01, ***p < 0.001, ns: not significant.

*Carbamylation vs glycation:* in the subtotal nephrectomy model, furosine content of skin type I collagen was increased (p < 0.01) in diabetic mice in comparison with control mice (2281 ± 799 µmol/mol Lys *vs* 710 ± 84 µmol/mol Lys, respectively) (Fig. 4A). This value was less elevated in CKD-diabetic mice: 1287 ± 314 µmol/mol Lys in CKD-diabetic mice (−44% in comparison with diabetic mice, p < 0.05). By contrast, no significant difference was observed between these two groups for CML concentrations, like in total extracts (Fig. 4B).

In the cyanate-consuming model, similar patterns as those obtained for total skin extracts were observed, showing a −25% decrease in furosine content in cy-diabetic mice in comparison with wa-diabetic mice (Fig. 4D). However, no differences in CML concentrations were observed between cy-diabetic and wa-diabetic groups (Fig. 4E). These findings show that the competitive effect of carbamylation on the early steps of glycation was also observed in collagen.

*Glycation vs carbamylation:* skin collagen HCit content was not significantly different between CKD and CKD-diabetic groups (Fig. 4C), showing the same results as those obtained in total extracts. Surprisingly, no significant increase of collagen carbamylation rate was observed in CKD mice when compared with control ones. In the cyanate-consuming mice model, results were similar to those obtained from skin total extracts: HCit concentrations were not lower in cy-diabetic conditions but even more elevated (3.3 fold increase, Fig. 4F).

## Discussion

Molecular aging of matrix proteins is an important process involved in tissue aging^14^, but remains incompletely understood, especially regarding the general and/or respective contributions of each NEPTM. Thus, the aim of this study was to determine whether NEPTMs could compete for the modification of matrix proteins, in order to better understand the role and the relative importance of each reaction. For that purpose, the competition between two among the most important NEPTMs (*i.e*. glycation and carbamylation) was evaluated in murine models. A previous study from our team has shown that these two reactions reciprocally compete *in vitro* but that only carbamylation was able to exert a potent competitive effect on glycation *in vivo* for the modification of hemoglobin and serum proteins^12^. In order to determine whether a similar competitive effect is observed with matrix proteins, carbamylation and glycation products were quantified in skin and aorta extracts. These tissues were selected because of their high content in long-lived matrix proteins. Previous data have shown that several lysine residues in skin type I collagen may be carbamylated and/or glycated during aging^4^. Aorta was also selected because vascular diseases are the most important long-term complications in patients with diabetes and CKD, two clinical contexts favouring glycation and/or carbamylation reactions.

The murine model associating CKD and diabetes used for reproducing the competition context *in vivo* was first validated using various biological parameters including blood glucose and urea. Then, the quantification of glycation and carbamylation products showed that glycation has no influence on the carbamylation process in this model, whereas carbamylation was able to exert a significant competitive effect by limiting the formation of Amadori products in CKD-diabetic mice in comparison with diabetic ones, these results being found in both analyzed tissues (Table 2). A similar conclusion had been drawn for circulating Amadori products such as HbA_1c_ and fructosamine^12^. The lack of reciprocal competitive effect could be explained by some differences between these two NEPTMs. During the glycation reaction, the formation of Amadori products is preceded by a reversible step corresponding to the formation of a Schiff base, whereas carbamylation corresponds to a one-step reaction. Thus, carbamylation can occur faster and in a rapidly irreversible way, even though it remains dependent on the time required for the dissociation of urea which is compulsory for isocyanic acid formation^15,16^.

CML results are also consistent with a competition of carbamylation on glycation but they are less evident, which may be explained by the preponderant role of oxidative stress in the formation of this AGE from Amadori products. Indeed, CKD promotes oxidative stress and may therefore participate in the formation of CML^17^. As a consequence, the diabetic nephropathy model used in this study is probably not perfectly suitable for evaluating the competiting effect of carbamylation on AGE formation. In addition, since AGEs are formed over a long period, extending the experiment beyond five weeks could allow to observe more significant effects. Finally, the results obtained for CML do not allow us to conclude on the influence of carbamylation on the formation of all type of AGEs because CML is only one among a large variety of AGEs. Other AGEs like pentosidine could be interesting to study but most of these compounds are formed as a result of complex additional reactions requiring more time than for CML formation, which would raise again the question of the experiment duration. Similarly, hydroimidazolone-type AGEs (*e.g*. methyl-glyoxal hydroimidazolone-1) formed from carbonyl derivatives, have not been evaluated in this study because their formation is not dependent on the binding of glucose on proteins, so that the CKD-diabetic mice model used here did not reproduce the suitable competition context.

Since the CKD-diabetic model exhibited some limitations, especially for the interpretation of CML concentrations, the experiments were repeated using another model, consisting in cyanate-fed mice. This model may be considered a more “pure” carbamylation model, making it possible to overcome side effects related to CKD like increased oxidative stress. The results obtained with this model confirmed that carbamylation is able to exert a competitive effect on glycation. However, this effect was surprisingly less important than in the case of the CKD-diabetic model. In addition, this model also has limitations because diabetic mice are polydipsic thereby enhancing the consumption of cyanate and thus increasing carbamylation level. This may explain the high values of HCit found in cy-diabetic mice tissues.

This competitive effect was then evaluated on matrix proteins, especially type I collagen because of its abundance in numerous tissues including skin. The interest in studying this protein was also based on its long half-life which makes it more subjected to NEPTMs. For that purpose, type I collagen was extracted from mice skin in the different groups. Quantification of glycation and carbamylation products showed similar results as in total extracts with a competition in favour of carbamylation. These results are consistent with our previous observations which showed that the proportion of potential carbamylation sites in human skin collagen was greater compared to potential glycation sites^4^. Additional experiments would be necessary to define the sites on which this competition takes place. Moreover, it seems that this competition only occurs when the number of available sites becomes limited (*i.e*. in hyperglycemic conditions) since the present results showed that no competitive effect was observed under normoglycemic conditions.

The modifications of matrix proteins by NEPTMs contribute to tissue aging at several levels: (*i*) mechanical properties of the tissues, (*ii*) turnover of matrix proteins and (*iii*) cell-matrix interactions. Indeed, numerous studies have shown that each NEPTM is able to alter the structural properties of matrix proteins which consequently impacts tissue mechanical properties^18,19^. For example, glycation has been shown to impact tendon mechanics by altering the molecular interactions at the fiber surface, thereby decreasing sliding between fibers^20^. By contrast, carbamylated type I collagen shows alterations in triple helix structure leading to a defect of fibrillogenesis^9^. These modifications also result in differences of sensitivity towards enzymatic proteolysis which contribute to matrix remodeling alterations and thereby to disturbance of tissue homeostasis^21,22^. Finally, glycation or carbamylation of key amino acid residues may disturb cell-extracellular matrix interactions^23,24^. As glycation and carbamylation sometimes lead to totally different effects on protein properties, the interest of studying their reciprocal competition and of determining which NEPTM is predominant is reinforced.

The complex set of interactions highlighted in this study makes uncertain the extrapolation of the results to clinical conditions. Indeed, both reactions exert deleterious effects but address various tissues or cells and involve distinct mechanisms. As long as the effects of both reactions on specific functions of targeted proteins are not fully established, it is difficult to speculate whether the predominance of carbamylation on glycation would eventually be beneficial or at least less harmful. On one hand, this competition could minimize glycation-induced adverse effects like protein crosslinking and inflammatory reactions linked to AGE-RAGE axis, and CDPs seem to be more easily cleared from the organism than AGEs^25^. However, most consequences of carbamylation are negative as demonstrated for example by its association with various pathologies such as cardiovascular diseases^2,26^. The ongoing elucidation of molecular mechanisms involved in CDP toxicity will provide new clues to the interpretation and further exploitation of these data for clinical purpose.

In conclusion, our data show that, when these two NEPTMs are enhanced at the same time (*e.g*. in CKD-diabetic conditions), carbamylation takes precedence over glycation for tissue protein modifications, and especially type I collagen, whereas glycation has been for long time considered the most important NEPTM. The existence of competition phenomena between NEPTMs should now be taken into account in future studies aiming at understanding the molecular mechanisms of tissue aging.
